# MetaPhat: Detecting and Decomposing Multivariate Associations From Univariate Genome-Wide Association Statistics

**DOI:** 10.3389/fgene.2020.00431

**Published:** 2020-05-15

**Authors:** Jake Lin, Rubina Tabassum, Samuli Ripatti, Matti Pirinen

**Affiliations:** ^1^Institute for Molecular Medicine Finland FIMM, Helsinki Institute of Life Science HiLIFE, University of Helsinki, Helsinki, Finland; ^2^Public Health, University of Helsinki, Helsinki, Finland; ^3^Broad Institute, Massachusetts Institute of Technology, Harvard University, Cambridge, MA, United States; ^4^Department of Mathematics and Statistics, University of Helsinki, Helsinki, Finland

**Keywords:** multivariate analysis, genotype phenotype correlation studies, feature selection, Bayesian information criteria, visualilzation, canonical correlation, multivariate GWAS, pheno- and genotypes

## Abstract

**Background:**

Multivariate testing tools that integrate multiple genome-wide association studies (GWAS) have become important as the number of phenotypes gathered from study cohorts and biobanks has increased. While these tools have been shown to boost statistical power considerably over univariate tests, an important remaining challenge is to interpret which traits are driving the multivariate association and which traits are just passengers with minor contributions to the genotype-phenotypes association statistic.

**Results:**

We introduce MetaPhat, a novel bioinformatics tool to conduct GWAS of multiple correlated traits using univariate GWAS results and to decompose multivariate associations into sets of central traits based on intuitive trace plots that visualize Bayesian Information Criterion (BIC) and *P*-value statistics of multivariate association models. We validate MetaPhat with Global Lipids Genetics Consortium GWAS results, and we apply MetaPhat to univariate GWAS results for 21 heritable and correlated polyunsaturated lipid species from 2,045 Finnish samples, detecting seven independent loci associated with a cluster of lipid species. In most cases, we are able to decompose these multivariate associations to only three to five central traits out of all 21 traits included in the analyses. We release MetaPhat as an open source tool written in Python with built-in support for multi-processing, quality control, clumping and intuitive visualizations using the R software.

**Conclusion:**

MetaPhat efficiently decomposes associations between multivariate phenotypes and genetic variants into smaller sets of central traits and improves the interpretation and specificity of genome-phenome associations. MetaPhat is freely available under the MIT license at: https://sourceforge.net/projects/meta-pheno-association-tracer.

## Introduction

Genome-wide association studies (GWAS) of common diseases and complex traits in large population cohorts have linked thousands of genetic variants to individual phenotypes. In emerging biobank studies as well as in some disease specific collections have focused on, for example, Type 2 diabetes (T2D) (Mahajan et al., 2018) or coronary artery disease (CAD) (Ripatti et al., 2016), multiple related quantitative traits are simultaneously available for genetic association studies. The statistical power in these discovery efforts can be boosted considerably by multivariate tests, which have become more practical through recent implementations that require only univariate summary statistics, such as MultiPhen (O’Reilly et al., 2012), TATES (van der Sluis et al., 2013), CONFIT (Gai and Eskin, 2018), MTAG (Turley et al., 2018), MTAR (Guo and Wu, 2019), and metaCCA (Cichonska et al., 2016). The merits of many of these methods are further discussed by Chung et al. (2019). Concretely, canonical correlation analysis (CCA) (Hotelling, 1936) is the direct extension of the correlation coefficient to identify linear associations between two sets of variables, and it has been successfully applied also to GWAS (Inouye et al., 2012). Moreover, metaCCA extended CCA to work directly from GWAS summary statistics (effect size estimates and standard errors) of related traits and studies. However, a remaining challenge is to interpret which traits are driving the multivariate association and which traits are just passengers contributing little to the association statistic. A successful identification of a subset of central traits for each associated variant can lead to new biological insights in studies of disease progression and heterogeneity. To address this important task, we have introduced MetaPhat (Meta-Phenotype Association Tracer), a novel method to efficiently and systematically:

1. identify and annotate significant variants via multivariate GWAS from univariate summary statistics using metaCCA;
2. perform decomposition by systematically tracing the traits of highest and lowest statistical importance to identify subsets of central traits at each associated variant;
3. plot the traces of trait decompositions and cluster the variants based on the ranking of the importance of traits.

## Materials and Methods

### Workflow

**FIGURE 1:**
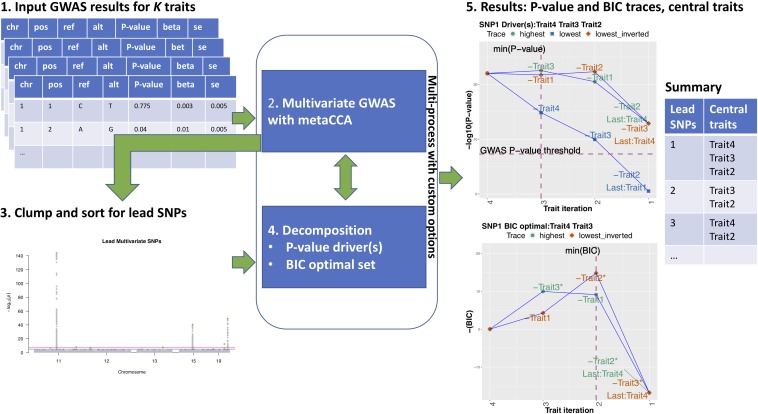
MetaPhat workflow **1.** GWAS results for K traits are accepted as input. **2.** After quality control and filtering, a multivariate GWAS is performed on the full model with all *K* traits using metaCCA via efficient multi-processing and chunking to reduce computation time. **3.** Lead SNPs are detected and sorted based on the leading canonical correlation/*P*-value and then clumped based on a user-specified window size. Custom variants can be added. **4.** Decomposition of chosen variants is performed through highest and lowest traces to find an optimal subset with a minimum BIC and driver traits based on the established *P*-value threshold. **5.** MetaPhat results include trace plots for *P*-values and BIC, univariate association statistics plots for all lead SNPs, cluster maps (shown in Figure 2), and a summary table listing central traits (union of drivers and optimal subset).

MetaPhat requires as input a set of related GWAS summary statistics from correlated traits. The program implements efficient multi-trait genome-wide association testing, identification of significant associations, and systematic tracing of trait subsets to identify the central traits that consist of a statistically optimal set of traits together with a set of driver traits. A workflow is shown in Figure 1. In steps one to three, genome-wide significant variants [*P* < 5e-8, the established genome-wide threshold in the field (Sherry et al., 2001; Pe’er et al., 2008)] were identified and were clumped into independent groups that are subsequently represented by the lead variant of each group (i.e., the variant with the smallest *P*-value). By default, two lead variants were defined as independent if their distance is higher than 1 million base pairs. At step four, we carried out the decompositions of multivariate association by starting from model with all K traits and removing one trait at a time until only one trait remains. We proceeded via two different strategies that we named the *highest trace* and the *lowest trace*. More specifically, starting from the model with all K traits, we tested all unique combinations of (K-1) traits to find the subset with the highest CCA statistic (lowest *P*-value) that we assigned to the highest trace and the subset with the lowest CCA statistic (highest *P*-value) that we assigned to the lowest trace. We continued both traces iteratively until only a single trait remained by always choosing the subset with the highest CCA statistic on the highest trace and the subset with the lowest CCA statistic on the lowest trace. Intuitively, at each step, the trait dropped on the highest trace was the trait that was best replaceable by the other traits in the model with respect to the genetic association considered. Analogously, at each step, the trait dropped on the lowest trace was the trait that was most irreplaceable by the other traits in the model with respect to the genetic association considered. Altogether, we evaluated K^2^ subsets out of all possible 2^*K*^ subsets while building these two traces. Base pair distances, GWAS *P*-value thresholds, and other program parameters could be updated using command-line arguments.

We used the two traces to identify central traits that are primarily responsible for the association with the variant as explained next.

### Evaluating Models

We used two quantities to evaluate models: CCA *P*-values and Bayesian Information Criterion (BIC; Schwarz, 1978). *P*-values allowed us to compare each association to the established “genome-wide significance threshold” of 5e-8 (Pe’er et al., 2008). By using the lowest trace, we could identify those traits without which the multivariate *P*-value is no longer genome-wide significant by simply collecting the traits that have been removed from the full model when the *P*-value on the lowest trace is first time above 5e-8. We call these traits the driver traits since they drive the association in the sense that without them the association does not anymore reach genome-wide significance and hence would not have been reported as a discovery in a GWAS. This definition of driver traits is based on a fixed P-value threshold, which is an established practice in the field, but does not claim any statistical optimality properties in terms of model comparison. Hence, to more rigorously compare models with different dimensionalities, we used BIC, which approximates the negative marginal likelihood of the model and thus penalizes for the model dimension (Schwarz, 1978). A lower BIC value suggests a statistically better description of the data. A subset of traits with minimum BIC would thus be the model of choice. We defined the optimal subset as the subset with the lowest BIC among all subsets on the highest trace and all subsets on the inverted lowest trace. The inverted lowest trace aggregates the traits that have been dropped on the lowest trace, and, in particular, includes the set of the driver traits as one of its subsets. Subsequently, we defined the central traits as the union of traits from the drivers and optimal BIC subset. MetaPhat traces and terms are summarized in Table 1.

**TABLE 1:** MetaPhat terminology.

Highest trace	Starting from the full model of K traits, we tested all unique combinations of (K-1) traits to find the subset with the highest CCA statistic (lowest *P*-value), and we iterated until *K* = 2. The goal was to drop most replaceable traits first.
Lowest trace	Starting from the full model of K traits, we tested all unique combinations of (K-1) traits to find the subset with the lowest CCA statistic (highest *P*-value), and we iterated until *K* = 2. The goal was to drop most irreplaceable traits first.
Inverted trace	Aggregates the traits that have been dropped on the lowest trace. The goal was to include the driver sets into the search space for the *optimal set*.
Drivers/driver traits	The traits that have been dropped on the lowest trace at the step where the multivariate *P*-value was for the first time no longer genome-wide significant. Interpretation: traits that make the multivariate association statistically significant.
Optimal set	The subset of traits that has the lowest BIC among subsets across all three traces. Interpretation: the set that is a statistically optimal description of the multivariate association.
Central traits	Union of drivers and optimal set. Interpretation: includes the important traits of the multivariate association.

### Computing *P*-Values and BIC From GWAS Summary Statistics

metaCCA outputs the first canonical correlation *r*_1_ between the genetic variant *x* and the set of *k* traits *y*_1_,…,*y*_*k*_ and computes the corresponding *P*-value (Clarke et al., 2011; Cichonska et al., 2016). In this case, the first canonical correlation *r*_1_ equals to the maximum correlation between the variant and any linear combination of the traits and hence is equal to the square root of the variance explained *R*^2^ from the linear regression of *x* on *y*_1_,…,*y*_*k*_. In general, the expression for BIC is

$$\text{BIC}=\log\left(n\right)\,k-2\,\hat{\log l\,k}$$

where *n* is the sample size, *k* is the number of parameters (here traits), and $\hat{\log l\,k}$ is the maximized log-likelihood. Next, we have shown how to use metaCCA output *r*_1_ to derive BIC from the maximized likelihood of the linear model written as a function of *R*^2^ = *r*_1_^2^.

Consider a linear model between a (mean-centered) variant *x* and (mean-centered) traits ***y*** = (*y*_1_,…,*y*_*k*_)*^*T*^*.

$$x=y^{T}\,\beta +\varepsilon =y_{1}\,\beta_{1}+\cdots +y_{k}\,\beta_{k}+\varepsilon ,\varepsilon ∼N\,\left(0,\sigma^{2}\right),$$

where we do not include the intercept parameter as its maximum likelihood estimate (MLE) is zero after mean-centering. The log-likelihood function is

$$\text{loglk}\,\left(\beta ,\sigma^{2}\right)=-\frac{n}{2}\,\text{log}\,\left(2\,\pi \right)-\frac{n}{2}\,\text{log}\,\left(\sigma^{2}\right)-\frac{{\left(x-y^{T}\,\beta \right)}^{T}\,\left(x-y^{T}\,\beta \right)}{2\,\sigma^{2}},$$

and MLEs are

β∧=(yT⁢y)-1⁢yT⁢x⁢and⁢σ2∧2=1n⁢((x-yT⁢β∧)T⁢(x-yT⁢β∧)).

Thus, the log-likelihood at maximum is

$$\,\hat{\log\text{lk}}=\log\mathrm{lk}\,\left(\hat{\beta },{\hat{\sigma }}^{2}\right)=-\frac{n}{2}\,\log\left(2\,\pi \right)-\frac{n}{2}\,\log\left(\hat{\sigma^{2}}\right)$$

$$-\frac{{\left(x-y^{T}\,\hat{\beta }\right)}^{T}\,\left(x-y^{T}\,\hat{\beta }\right)}{\hat{\sigma^{2}}}-\frac{n}{2}\,\log\left(2\,\pi \right)-\frac{n}{2}\,\log\left(\hat{\sigma^{2}}\right)-\frac{n}{2}$$

$$R^{2}=x^{T}\,x-\frac{{\left(x-y^{T}\,\hat{\beta }\right)}^{T}\,\left(x-y^{T}\,\hat{\beta }\right)}{x^{T}\,x}=1-\frac{\hat{\sigma^{2}}}{\hat{\sigma_{0}^{2}}},$$

that  is,

$$\frac{\hat{\sigma^{2}}}{\hat{\sigma_{0}^{2}}}=1-R^{2}\,\mathrm{where}\,\hat{\sigma_{0}^{2}}=\mathrm{var}\,\left(x\right).$$

Hence, the logarithm of the likelihood ratio between the MLE and the null model can be written as

$$\log\text{LR}=\hat{\log\text{lk}}-\log\mathrm{lk}\,\left(0,\hat{\sigma_{0}^{2}}\right)=-\frac{n}{2}\,\log\frac{\hat{\sigma^{2}}}{\hat{\sigma_{0}^{2}}}=1-\frac{n}{2}\,\log\left(1-R^{2}\right).$$

Hence, we have that, for an additive constant c=-2⁢loglk⁢(0,σ02∧2),

$$\text{BIC}=k\,\log\left(n\right)-2\,\left(\hat{l\,o\,g\,l\,k}\right)=k\,\log\left(n\right)+n\,\log\left(1-R^{2}\right)+c,$$

which is possible to compute directly from the metaCCA output for models with at least two traits up to an additive constant *c.* Since *c* does not depend on the model dimension, we can ignore it in the BIC calculation, when we are only interested in the differences in BIC between models.

Finally, for a single-trait model, *R*^2^ can be computed directly from the univariate GWAS summary statistics as

$$R^{2}=\frac{1}{\left(1+n/z^{2}\right)}\,\text{where}\,z=\frac{\mathrm{GWAS}\,\mathrm{effect}}{\mathrm{standard}\,\mathrm{error}},$$

which can be plugged in the BIC formula above to yield BIC for the single-trait model.

### Implementation and Output

MetaPhat is written in Python (compatible for 2.7 and 3+) and requires R (3.4+) for plotting. The command-line based program has been tested on multiple operating systems and cloud images. Library requirements and command options are further described in Supplementary Table S1, and test data are accessible from the project page: https://sourceforge.net/projects/meta-pheno-association-tracer.

MetaPhat outputs tabular text files and several plots. A summary result file contains, for each chosen variant, the driver traits and the optimal subset with their *P*-value and BIC statistics. For each variant, trace plots using *P*-values and BIC are generated, showing the highest trace, the lowest trace and the inverted lowest trace. In addition, the univariate *P*-values and directions of effects for each trait are also plotted. The estimated phenotype correlation matrix, clustered heatmaps of trait importance for the chosen variants and a similarity between variants using trait rankings on the lowest trace are produced. Optionally, intermediate statistics during the decomposition can be plotted to get a more detailed view of the decomposition process.

### Materials

Our lipidomics data set consisted of the univariate GWAS results of 21 correlated lipid species with polyunsaturated fatty acids that were reported to exhibit high heritability (Tabassum et al., 2019) and showed high correlation (Supplementary Figure S2). These results originated from 2,045 Finnish subjects with imputed genotypes available at ∼8.5 million SNPs. The arbitrarily assigned lipid species identifiers along with their class names and fatty acid chemical properties are listed in Table 2A. To further validate MetaPhat, we processed summary statistics from four basic lipids [high-density lipoprotein (HDL) cholesterol, low-density lipoprotein (LDL) cholesterol, triglycerides (TG), and total cholesterol (TC)] conducted by the Global Lipids Genetics Consortium (GLGC) (Willer et al., 2013; Zhu et al., 2018), and these are listed in Table 2B. With the GLGC data set our aim was to compare MetaPhat results with univariate results reported by GLGC for all variants reported to be significantly associated with two or more traits by GLGC.

**TABLE 2:** Lipid traits used in MetaPhat analysis.

(A) PLASMA LIPIDOMICS						

Identifier	Lipid class	Lipid species	QC’d variants	HDL corr.	LDL corr.	TG corr.
CE14	Cholesteryl ester	*CE*(20:4;0)	8,711,715	0.032	0.464	0.251
CE15	Cholesteryl ester	*CE*(20:5;0)	8,711,715	0.067	0.396	0.188
CE17	Cholesteryl ester	*CE*(22:6;0)	8,711,665	0.107	0.394	0.107
LPC8	Lysophospatidylcholines	*LPC*(20:4;0)	8,710,151	0.011	–0.124	–0.083
LPC9	Lysophospatidylcholines	*LPC*(22:6;0)	8,694,250	0.114	–0.015	–0.118
LPE5	Lysophosphatidylethanolamine	*LPE*(20:4;0)	8,710,162	0.077	–0.077	0.073
LPE6	Lysophosphatidylethanolamine	*LPE*(22:6;0)	8,711,037	0.235	0.005	0.041
PC17	Phosphatidylcholine	*PC*(16:0;0−20:4;0)	8,711,715	0.120	0.115	0.361
PC18	Phosphatidylcholine	*PC*(16:0;0−20:5;0)	8,711,533	0.126	0.196	0.248
PC29	Phosphatidylcholine	*PC*(17:0;0−20:4;0)	8,704,982	0.113	0.138	0.250
PC36	Phosphatidylcholine	*PC*(18:0;0−20:4;0)	8,711,715	0.033	0.190	0.336
PC37	Phosphatidylcholine	*PC*(18:0;0−20:5;0)	8,751,062	0.061	0.242	0.243
PC46	Phosphatidylcholine	*PC*(18:1;0−20:4;0)	8,711,715	0.240	0.105	0.214
PC21	Phosphatidylcholine	*PC*(16:0;0−22:6;0)	8,711,715	0.154	0.204	0.219
PCO7	Phosphatidylcholine-ether	*PC*−*O*(16:0;0−20:4;0)	8,711,715	0.081	0.194	0.076
PCO23	Phosphatidylcholine-ether	*PC*−*O*(18:0;0−20:4;0)	8,711,560	0.187	0.115	–0.154
PCO29	Phosphatidylcholine-ether	*PC*−*O*(18:1;0−20:4;0)	8,710,292	0.198	0.115	–0.086
PE7	Phosphatidylethanolamine	*PE*(18:0;0−20:4;0)	8,707,361	–0.027	0.028	0.585
PEO3	Phosphatidylethanolamine-ether	*PE*−*O*(16:1;0−20:4;0)	8,706,846	0.083	0.198	0.154
PEO11	Phosphatidylethanolamine-ether	*PE*−*O*(18:2;0−20:4;0)	8,693,147	0.148	0.238	0.099
PI9	Phosphatidylinositol	*PI*(18:0;0−20:4;0)	8,711,715	–0.026	0.231	0.460

**(B) GLGC LIPIDS**

**Identifier**	**Lipid class**	**QC’d variants**	**Sample size**

HDL	High-density lipoprotein cholesterol	2,343,025	95,129
LDL	Low-density lipoprotein cholesterol	2,271,091	90,421
TC	Total cholesterol	2,341,292	95,537
TG	Triglycerides	2,286,633	91,598

****(A)****Polyunsaturated lipid species with acyl chains- C20:4 (14 lipids), C20:5 (3 lipids), and C22:6 (4 lipids) measured for 2,045 individuals (Tabassum et al., 2019). After quality control (QC), a total of 8,576,290 variants were available for all 21 traits. Correlations to basic lipids HDL, LDL, and TG are also shown.****(B)****Four basic lipids from GLGC (Willer et al., 2013). After quality control, a total of 2,267,285 variants were available for all four traits.**

## Results

Using the lipidomics data sets with GWAS summary statistics from the 21 polyunsaturated lipids, MetaPhat found seven independent lead variants after clumping the 415 variants exceeding the standard GWAS *P*-value threshold of 5e-8 within a window of 1 Mb. Table 3 lists these variants along with their gene annotation, multivariate *P*-value, and central traits. MetaPhat has strongly reduced the multivariate association for all seven variants into smaller and more specific groups of central traits.

**TABLE 3:** MetaPhat results of the 7 lead variants from the multivariate analyses of the lipidomics data.

Variant/Gene	Samples missing	*P*-value all traits	Driver trait(s)	*P*-value without drivers	BIC optimal subset	*P*-value BIC optimal subset	Central traits
*rs174567/FADS2	1.3%	2.40e–145	PC36, CE14, PC17, LPC8, PEO11, PEO3, LPE5, PC21, PC46, PC29, CE15, PC37, PC18, PCO7, PCO29, PCO23, PI9, PE7	1.95e–05	CE15, LPC8, PC17, PC21, PC36, PC46, PE7, PEO11, PI9	2.10e–146	PC36, CE14, PC17, LPC8, PEO11, PEO3, LPE5, PC21, PC46, PC29, CE15, PC37, PC18, PCO7, PCO29, PCO23, PI9, PE7
*rs66505542/BUD13	0.1%	1.55e–08	PI9	3.39e–04	PI9, LPC9, PC36	3.27e–12	PI9, LPC9, PC36
rs146327691/SLCO1A2_ UTR	1.2%	4.27e–08	LPE5	1.91e–06	LPE5, LPC9, LPE6, PE7	5.60e–11	LPE5, LPC9, LPE6, PE7
rs188167837/ENSG00 000200733_UTR_13KB	1.0%	2.95e–08	PC17	7.59e–05	PC17, CE14, CE17, PC21	4.64e–09	PC17, CE14, CE17, PC21
*rs261290/ALDH1A2	0.6%	2.51e–40	PE7	2.04e–07	PE7, CE15, PC17, PCO29, PI9	1.37e–46	PE7, CE15, PC17, PCO29, PI9
*rs7412/APOE	0%	4.17e–13	CE14, PCO23	1.82e–06	CE14, PCO23, PC36	5.79e–18	CE14, PCO23, PC36
rs8736/MBOAT7	23.6%	9.12e–50	PI9	5.89e–02	PI9, LPE6, PC36, PE7	1.25e–81	PI9, LPE6, PC17

The lipid trait class names and acyl chain properties are listed in Table 2A. *Variant region reported as significant for basic lipids by GLGC (Willer et al., 2013).

**FIGURE 2:**
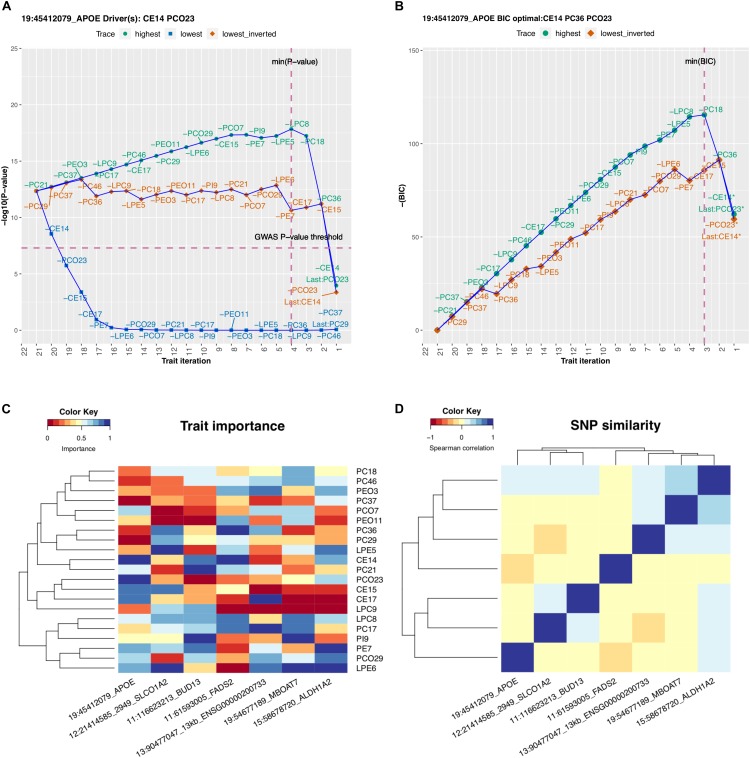
MetaPhat results using multivariate lipidomics data. **(A)** Trace plot of rs7412 identifies CE14 and PCO23 as the driver traits. **(B)** CE14, PC36, and PCO23 form the optimal subset as defined by minimum BIC (highest negative BIC). **(C)** Trait importance map of each SNP is the rank on the lowest trace where the rankings are transformed to the range of 0 and 1 values, with darker blue shades representing the most important traits of the multivariate association. **(D)** SNP similarity based on the rank correlation on the lowest trace.

We considered in more detail rs7412, which is a missense variant in the *APOE* gene and is known for its effect on LDL, as reported, for example, in the GLGC analysis (Willer et al., 2013). With the lipidomics data, this variant would not have been identified from any of the 21 univariate GWAS as the smallest univariate *P*-value was 1.1e-4 (trait PCO23, Supplementary Figure S3.6). On contrary, the multivariate GWAS by MetaPhat clearly highlighted this variant associated with the multivariate lipidomics (*P* = 4.2e-13) and further determined that the association was driven by CE14 and PCO23 (*P*-value after excluding these driver traits is 1.8e-06). The BIC-optimal subset for this variant extended the drivers by one additional trait and included CE14, PC36, and PCO23, which form the central traits. The trace plots for rs7412 are shown in Figure 2A (*P*-values for defining driver traits) and Figure 2B (BIC for defining optimal subset).

Variants rs66505542 near *BUD13* and rs261290 near *ALDH1A2* both have only one driver trait (PI9 for *BUD13* and PE7 for *ALDH1A2*) and three or five central traits (Table 3). Earlier, the *APOA1* variant rs964184 within 100 kb of rs66505542 has been reported to be associated with TG (lead trait, *P* = 7.0e-224), TC, HDL, and LDL in GLGC data and rs66505542 itself with several cell phenotypes (platelet count, red cell distribution width, sum of eosinophil and basophil counts) in the GWAS catalog, while rs261290 has been reported to be associated with HDL (lead trait, *P* = 1.0e-188), TC, and TG in GLGC data (mapped to *LIPC* gene) and with HDL in the GWAS catalog.

A very different picture emerges for rs174567 near *FADS1/2* since its 18 central traits show its wide effects across the lipidomics traits studied here. Previously reported *FADS1/2* associations are with all lipid traits (TG lead trait, *P* = 7.0e-38) in GLGC data and with metabolite measurements and gallstones in the GWAS catalog.

Trait importance map that clusters each variant based on the lowest trace is shown in Figure 2C and the similarity of the variants as measured by rank correlation of the traits on the lowest trace is shown in Figure 2D. The trace plots for the other six variants than rs7412 are shown in Supplementary Figure S1.

### Validation and Global Lipids Genetics Consortium

We processed the Global Lipids Genetics Consortium (GLGC) GWAS study for four plasma lipids (HDL, LDL, TC, and TG, as listed in Table 2B). These correlated traits along with large sample sizes and available summary files are suitable for MetaPhat GWAS and decomposition. We focused on the 13 variants reported by GLGC to have associations with three or more lipid traits (Supplementary Tables S2 and S3 from Willer et al., 2013). In Table 4, we validated that at 12 out of the 13 variants the same associations are confirmed by MetaPhat’s central traits. The only discordance was at rs6831256 (*DOK7*) where we found TC and TG as central traits compared to previously reported univariate associations with TC, TG, and LDL. As TC and LDL are highly correlated, it is understandable that the smaller dimension of the set TC, TG, may in some analyses be preferred over the set that also includes LDL. In Supplementary Table S2, we further report high concordance between our central traits and GLGC variants found associated with two or more standard lipids.

**TABLE 4:** MetaPhat detection of driver and optimal lipid sets for 13 variants reported to be associated with at least three lipids by GLGC (12).

Gene	Variant Chr:Pos	GLGC associated lipids	GLGC lead *P*-value	MetaPhat all traits *P*-value	MetaPhat driver(s)	Without driver (s) *P*-value	BIC optimal set	Central traits
		**HDL lead**						

*PIGV*-*NR0B2*	rs12748152 chr1:27138393	HDL LDL TG	1e–15	2.8e–23	HDL LDL TG	3.0e–06	HDL LDL	HDL LDL TG
*PPP1R3B*	rs9987289 chr8:9183358	HDL LDL TC	2e–41	1.6e–76	HDL TC LDL	1.0e–04	HDL LDL	HDL LDL TC
*LIPC* (*ALDH1A2)*	rs1532085 chr15:58683366	HDL TC TG	1e–188	0	HDL TC TG	6.4e–01	HDL TC TG	HDL TC TG
*CETP*	rs3764261 chr16:56993324	ALL	1e–769	0	ALL	NA	HDL LDL TG	ALL

		**LDL lead**						

*MIR148A*	rs4722551 7:25991826	LDL TG TC	4e–14	2.5e–24	TG LDL TC	2.0e–02	LDL TG	LDL TG TC
*APOE*	rs4420638 19:45422946	ALL	2e–178	6.3e–210	ALL	NA	LDL HDL TC	ALL

		**TC lead**						

*TIMD4*	rs6882076 5:156390297	TC LDL TG	5e–41	1.3e–49	TG TC LDL	6.9e–01	TC TG	TC LDL TG
*CILP2*	rs10401969 19:19407718	TC TG LDL	4e–77	1.3e–138	TG TC LDL	1.0e–01	TC TG	TC TG LDL

		**TG lead**						

*LRPAP1* (*DOK7*)	rs6831256 4:3473139	TG TC LDL	2e–12	6.3e–16	TG TC	1.0e–07	TG TC	TG TC
*ANGPTL3*	rs2131925 1:63025942	TG LDL TC	3e–74	7.8e–157	TG LDL TC	9.5e–05	TG TC HDL	ALL
*TRIB1*	rs2954029 8:126490972	ALL	1e–107	1.6e–148	ALL	NA	TG TC LDL	ALL
*FADS123*	rs174546 11:61569830	ALL	7e–38	1.3e–104	ALL	NA	ALL	ALL
*APOA1*	rs964184 11:116648917	ALL	7e–224	7.9e–264	ALL	NA	TG TC	ALL

**We confirmed that the vast majority of the MetaPhat central traits are either the same or a subset of the reported GLGC associated lipids (11/13 for driver traits, 12/13 for BIC).**

### Performance

For computing the test statistic, MetaPhat uses metaCCA that, for a single SNP, has previously been shown to reliably estimate the results of standard CCA applied to individual level data (canoncorr function in Matlab) (Cichonska et al., 2016). Additionally, we also empirically validated MetaPhat multivariate findings with GLGC results.

MetaPhat considerably cuts down the computational demands of comprehensive subset testing. With K traits, there are 2^*K*^-1 non-empty subsets that have quickly become infeasible to systematically assess, while MetaPhat only considers about K^2^ models. For example, in our example with *K* = 21 traits, the gain in performance is about 4,700-fold compared to the complete subset testing. To further increase performance and usability, we have implemented flexibility for multi-thread processing to enable high performance and memory efficiency. On a moderate Google cloud image (16 vCPUs, 8 GB), the complete MetaPhat workflow for our lipidomics analysis, containing 21 lipids and 8.5 million SNPs, was completed in less than 2.5 h (143 min). Using 10 processors and 9 gigabytes of memory, the GLGC job with the four basic lipids and 2.4 million imputed SNPs completed in 24 min. MetaPhat also allows decomposition and plotting of custom SNPs. For example, the custom analysis of the 13 GLGC variants associated with three or more traits, shown in Table 4, was run again on existing GLGC MetaPhat results, and decomposition and plotting took only 2 min. We note that the run time could be longer on shared servers but also substantially shorter using more powerful dedicated cloud images.

## Discussion

It is expected that a particular genetic variant may affect only a subset of related biomarkers that are risk factors of complex disorders, such as T2D or coronary heart disease. We implemented MetaPhat to systematically decompose and visualize statistically significant multivariate genome-phenome associations into a smaller group of central traits, based only on univariate GWAS summary statistics. We are not aware of comparable software to MetaPhat that would automatically carry out multivariate GWAS and identify central traits for the associations from summary statistics. ASSET (Bhattacharjee et al., 2012) aims to find the best trait subsets within a pool of multiple studies and has been applied particularly for case-control studies. MTAG (Turley et al., 2018) can be applied to GWAS results of multiple related traits and overlapping samples, but its aim is to improve the accuracy of the univariate effect sizes by using the information from correlated traits rather than decomposing the multivariate association to individual traits.

In our results from an analysis of 21 lipidomics traits, we demonstrated that the *APOE* association (rs7412) benefited from multivariate testing (driven by CE14 and PCO23 traits), as the univariate *P*-value was insignificant (*P* > 1e-4) across all 21 GWAS traits (shown in Supplementary Figure S3.6), but multivariate *P*-value was low (*P* < 5e-13). This variant is known to have a strong effect on LDL, and Table 2 shows that CE14 has the highest correlation with LDL (0.464). The other two central traits of this variant, PCO23 and PC36, did not have any correlation to basic lipids larger than 0.20 in absolute magnitude.

Table 3 lists the multivariate results including which four of these seven variants were previously reported by GLGC as associated with at least one of the four basic lipids. The other three variants also have some nearby variants that have been reported in the GWAS catalog (Buniello et al., 2019). First, rs8736 in *MBOAT7* has been previously reported to be associated with human blood metabolites (Shin et al., 2014) as well as alcohol related cirrhosis of the liver (Buch et al., 2015). Second, variants in the region of rs146327691, near the *SLCO1A2* gene, have been previously reported for response to serum metabolites (Krumsiek et al., 2012) and, interestingly, also for response to statins (Ho et al., 2006; Carr et al., 2019). Lastly, variants in the region of rs188167837 have been previously identified to be associated with nasopharyngeal carcinoma (Su et al., 2013). Additionally, MetaPhat decomposed most variants to substantially smaller sets of central traits than the full set of 21 traits, which can provide new biological insight regarding the variants identified. On the other hand, the essential role of *FADS2* gene region in regulating unsaturation in fatty acids was clearly reflected in MetaPhat results, as we observed as many as 18 central traits at the lead variant. Provided that the exact mechanistic roles of polyunsaturated lipids toward heart disease (Teslovich et al., 2010; Malovini et al., 2016; Pizzini et al., 2017) are under active investigation, our findings warrant further evaluation. We further confirmed good concordance (60/67, Supplementary Table S2) with MetaPhat central traits with respect to the earlier reported GLGC associations with two or more standard lipids, and excellent concordance (12/13) with the associations with three or more standard lipids.

MetaPhat optimal subsets are derived from the minimum BIC score representing the model that best describes the data when we account for both the model fit and the model dimension. Qualitatively BIC statistic is similar to the widely-used AIC (Akaike, 1973) statistic, but BIC quantitatively differs from AIC by favoring smaller dimensions, which also improves the interpretation of the optimal models. As intuitively expected, and as seen in Table 3, the driver traits tend to be members of the optimal set although they do not always agree, since the driver traits are defined by a GWAS-specific criterion of *P*-value threshold 5e-8, which does not need to coincide with the optimal subset chosen by a more statistically justified BIC criterion.

Our software implements flexible parameters for custom multi-thread chunking to enable high performance, genome-wide, multi-trait meta-analysis while integrating metaCCA for multivariate testing followed by systematic decomposition of traits. Thus, a limitation of MetaPhat is that it relies on metaCCA, but other multivariate GWAS algorithms could also be used provided that these methods can work with univariate GWAS results as inputs and produce suitable metrics that can be used to derive the model comparison statistics. With regard to false positives, we used the standard GWAS cutoff (*P* = 5e-8), as carried out only a single multivariate GWAS to pick the lead variants. This cutoff can be adjusted according to the preferences of the users. MetaPhat also optionally allows the running of metaCCA+ (Cichonska et al., 2016) shown to protect against false positives via shrinkage that adds robustness to the analysis.

Finally, we remind the reader that MetaPhat decompositions are sequential, dropping one trait at a time, and hence are not guaranteed to produce the globally optimal subset. Additionally, for highly correlated traits, such as LDL and total cholesterol, the choice of which one is dropped first may not be completely robust to small changes in data.

The ability of MetaPhat to identify and visualize central traits will also be valuable in supporting efforts and pipelines (Fatumo et al., 2019) comparing results between univariate and multivariate associations as well as in studies that aim to increase specificity of multi-trait associations. We also expect that the multi-phenotype clustering results of MetaPhat can assist researchers investigating disease subtypes.

## Data Availability Statement

All datasets generated for this study are included in the article/Supplementary Material.

## Author Contributions

MP, SR, and JL conceived the project. MP developed the theory. JL implemented and tested the method. RT assisted with the testing. JL and MP draft the manuscript. All authors provided critical feedbacks and important contributions to the final manuscript.

## Conflict of Interest

The authors declare that the research was conducted in the absence of any commercial or financial relationships that could be construed as a potential conflict of interest.

## References

[B1] Akaike H. (1973). “Information theory and an extension of the maximum likelihood principle,” in *Proceedings of the 2nd International Symposium on Information Theory*, eds Petrov B. N. Csáki F. (Budapest: Akadémiai Kiadó), 267–281.

[B2] Bhattacharjee S. Rajaraman P. Jacobs K. B. Wheeler W. A. Melin B. S. Hartge P. (2012). A subset- based approach improves power and interpretation for the combined analysis of genetic association studies of heterogeneous traits. *Am. J. Hum. Genet.* 90 821–835. 10.1016/j.ajhg.2012.03.015 22560090 PMC3376551

[B3] Buch S. Stickel F. Trépo E. Way M. Herrmann A. Nischalke H. D. (2015). A genome-wide association study confirms PNPLA3 and identifies TM6SF2 and MBOAT7 as risk loci for alcohol-related cirrhosis. *Nat. Genet.* 47 1443–1448. 10.1038/ng.3417 26482880

[B4] Buniello A. MacArthur J. A. L. Cerezo M. Harris L. W. Hayhurst J. Malangone C. (2019). The NHGRI-EBI GWAS catalog of published genome-wide association studies, targeted arrays and summary statistics 2019. *Nucleic Acids Res.* 47 D1005–D1012. 10.1093/nar/gky1120 30445434 PMC6323933

[B5] Carr D. F. Francis B. Jorgensen A. Zhang E. Chinoy H. Heckbert S. R. (2019). Genomewide association study of statin-induced myopathy in patients recruited using the UK clinical practice research datalink. *Clin. Pharmacol. Ther.* 106 1353–1361. 10.1002/cpt.1557 31220337 PMC6896237

[B6] Chung J. Jun G. R. Dupuis J. (2019). Comparison of methods for multivariate gene-based association tests for complex diseases using common variants. *Eur. J. Hum. Genet.* 27 811–823. 10.1038/s41431-018-0327-8 30683923 PMC6461986

[B7] Cichonska A. Rousu J. Marttinen P. Kangas A. J. Soininen P. Lehtimäki T. (2016). metaCCA: summary statistics-based multivariate meta-analysis of genome-wide association studies using canonical correlation analysis. *Bioinformatics* 32 1981–1989. 10.1093/bioinformatics/btw052 27153689 PMC4920109

[B8] Clarke G. M. Anderson C. A. Pettersson F. H. Cardon L. R. Morris A. P. Zondervan K. T. (2011). Basic statistical analysis in genetic case-control studies. *Nat. Protoc.* 6 121–133. 10.1038/nprot.2010.182 21293453 PMC3154648

[B9] Fatumo S. Carstensen T. Nashiru O. Gurdasani D. Sandhu M. Kaleebu P. (2019). Complimentary methods for multivariate genome-wide association study identify new susceptibility genes for blood cell traits. *Front. Genet.* 10:334 10.3389/fgene.2019.00334 PMC6497788 31080455

[B10] Gai L. Eskin E. (2018). Finding associated variants in genome-wide association studies on multiple traits. *Bioinformatics* 34 i467–i474. 10.1093/bioinformatics/bty249 29949991 PMC6022769

[B11] Guo B. Wu B. (2019). Integrate multiple traits to detect novel trait–gene association using GWAS summary data with an adaptive test approach. *Bioinformatics* 35 2251–2257. 10.1093/bioinformatics/bty961 30476000 PMC6596889

[B12] Ho R. H. Tirona R. G. Leake B. Glaeser H. (2006). Drug and bile acid transporters in rosuvastatin hepatic uptake: function, expression, and pharmacogenetics. *Gastroenterology* 130 1793–1806. 10.1053/j.gastro.2006.02.034 16697742

[B13] Hotelling H. (1936). Relations between two sets of variates. *Biometrika* 28 321–377. 10.1093/biomet/28.3-4.321

[B14] Inouye M. Ripatti S. Kettunen J. Leo-Pekka L. Oksala N. Laurila P. (2012). Novel loci for metabolic networks and multi-tissue expression studies reveal genes for atherosclerosis. *PLoS Genet.* 8:e1002907. 10.1371/journal.pgen.1002907 22916037 PMC3420921

[B15] Krumsiek J. Suhre K. Evans A. M. Matthew W. Robert P. M. Michael V. (2012). Mining the unknown: a systems approach to metabolite identification combining genetic and metabolic information. *PLoS Genet.* 8:e1003005. 10.1371/journal.pgen.1003005 23093944 PMC3475673

[B16] Mahajan A. Taliun D. Thurner M. Robertson N. R. Torres J. M. Rayner N. W. (2018). Fine-mapping type 2 diabetes loci to single-variant resolution using high-density imputation and islet-specific epigenome maps. *Nat. Genet.* 50 1505–1513. 10.1038/s41588-018-0241-6 30297969 PMC6287706

[B17] Malovini A. Bellazzi R. Napolitano C. Guffanti G. (2016). Multivariate methods for genetic variants selection and risk prediction in cardiovascular Diseases. *Front. Cardiovasc. Med.* 3:17. 10.3389/fcvm.2016.00017 27376073 PMC4896915

[B18] O’Reilly P. F. Hoggart C. J. Pomyen Y. Federico C. F. Paul C. Marjo-Riitta E. (2012). MultiPhen: joint model of multiple phenotypes can increase discovery in GWAS. *PLoS One* 7:e34861. 10.1371/journal.pone.0034861 22567092 PMC3342314

[B19] Pe’er I. Yelensky R. Altshuler D. Daly M. J. (2008). Estimation of the multiple testing burden for genomewide association studies of nearly all common variants. *Genet. Epidemiol.* 32 381–385. 10.1002/gepi.20303 18348202

[B20] Pizzini A. Lunger L. Demetz E. Hilbe R. Weiss G. Ebenbichler C. (2017). The role of omega-3 fatty acids in reverse cholesterol transport: a review. *Nutrients* 9:1099. 10.3390/nu9101099 28984832 PMC5691715

[B21] Ripatti P. Rämö J. T. Söderlund S. Surakka I. Matikainen N. Pirinen M. (2016). The contribution of GWAS loci in familial dyslipidemias. *PLoS Genet.* 12:e1006078. 10.1371/journal.pgen.1006078 27227539 PMC4882070

[B22] Schwarz G. E. (1978). Estimating the dimension of a model. *Ann. Statist.* 6 461–464. 10.1214/aos/1176344136

[B23] Sherry S. T. Ward M. H. Kholodov M. Baker J. Phan L. Smigielski E. M. (2001). dbSNP: the NCBI database of genetic variation. *Nucleic Acids Res.* 29 308–311. 10.1093/nar/29.1.308 11125122 PMC29783

[B24] Shin S. Y. Fauman E. B. Petersen A. K. Krumsiek J. Santos R. Huang J. (2014). An atlas of genetic influences on human blood metabolites. *Nat. Genet.* 46 543–550. 10.1038/ng.2982 24816252 PMC4064254

[B25] Su W. H. Yao J. Shugart Y. Chang K. P. Tsang N. M. Tse K. P. (2013). How genome-wide SNP-SNP interactions relate to nasopharyngeal carcinoma susceptibility. *PLoS One* 8:e83034. 10.1371/journal.pone.0083034 24376627 PMC3871583

[B26] Tabassum R. Rämö J. T. Ripatti P. Jukka T. K. Mitja K. Karjalainen J. (2019). Genetic architecture of human plasma lipidome and its link to cardiovascular disease. *Nat. Commun.* 10:4329. 10.1038/s41467-019-11954-8 31551469 PMC6760179

[B27] Teslovich T. M. Musunuru K. Smith A. V. Edmondson A. C. Stylianou I. M. Koseki M. (2010). Biological, clinical and population relevance of 95 loci for blood lipids. *Nature* 466 707–713. 10.1038/nature09270 20686565 PMC3039276

[B28] Turley P. Walters R. K. Maghzian O. Okbay A. Lee J. J. Fontana M. A. (2018). Multi-trait analysis of genome-wide association summary statistics using MTAG. *Nat. Genet.* 50 229–237. 10.1038/s41588-017-0009-4 29292387 PMC5805593

[B29] van der Sluis S. Posthuma D. Dolan C. V. (2013). TATES: efficient multivariate genotype-phenotype analysis for genome-wide association studies. *PLoS Genet.* 9:e1003235. 10.1371/journal.pgen.1003235 23359524 PMC3554627

[B30] Willer C. J. Schmidt E. M. Sengupta S. Peloso G. M. Gustafsson S. Kanoni S. (2013). Discovery and refinement of loci associated with lipid levels. *Nat. Genet.* 45 1274–1283. 10.1038/ng.2797 24097068 PMC3838666

[B31] Zhu Z. Anttila V. Smoller J. W. Lee P. H. (2018). Statistical power and utility of meta-analysis methods for cross-phenotype genome-wide association studies. *PLoS One* 13:e0193256. 10.1371/journal.pone.0193256 29494641 PMC5832233

