# Supplementary material for: MetaPhat: Detecting and Decomposing Multivariate Associations From Univariate Genome-Wide Association Statistics
Source: Front Genet. 2020 May 15;11:431. doi: 10.3389/fgene.2020.00431 (PMC7242752; doi:10.3389/fgene.2020.00431)
Supplement: Supplementary file 1 [file Data_Sheet_1.pdf]

Title: MetaPhat: Detecting and decomposing multivariate associations from univariate genome-wide association statistics

Jake Lin[1*], Rubina Tabassum[1], Samuli Ripatti[1,2,3], Matti Pirinen[1,2,4*]

1. Institute for Molecular Medicine Finland FIMM, HiLIFE, University of Helsinki, Helsinki, Finland.
2. Public Health, University of Helsinki, Helsinki, Finland.
3. Broad Institute of Massachusetts Institute of Technology and Harvard University, Cambridge, MA, USA.
4. Department of Mathematics and Statistics, University of Helsinki, Helsinki, Finland.

*Corresponding authors: jake.lin@helsinki.fi and matti.pirinen@helsinki.fi

Supplemental data

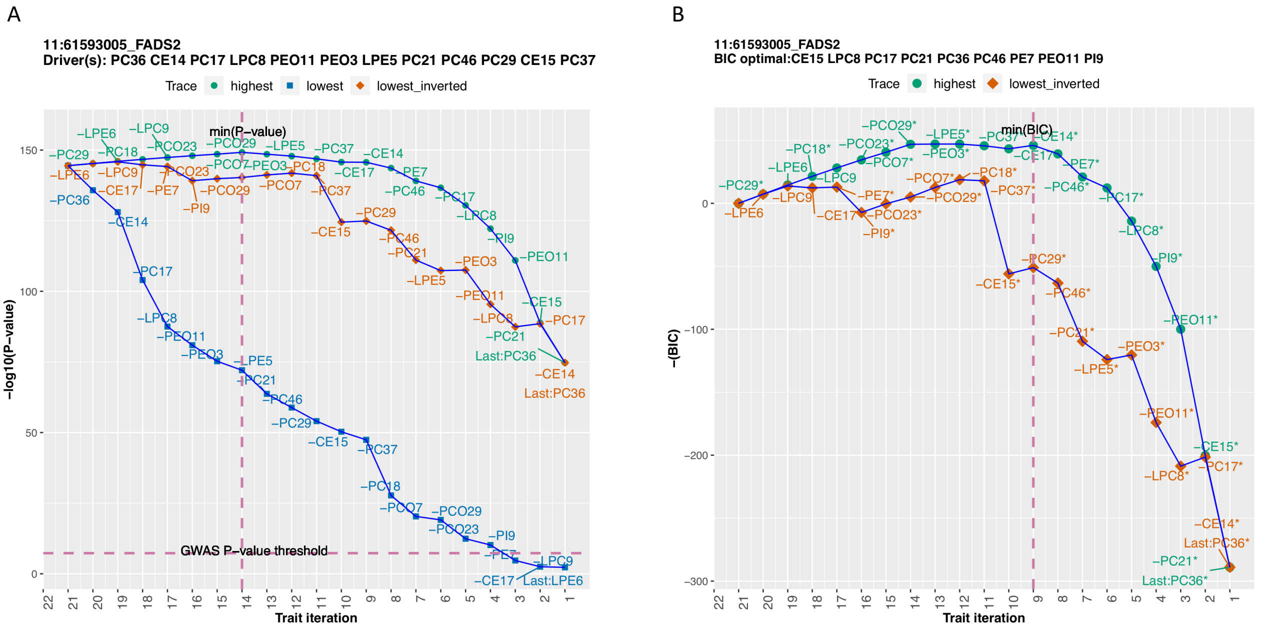

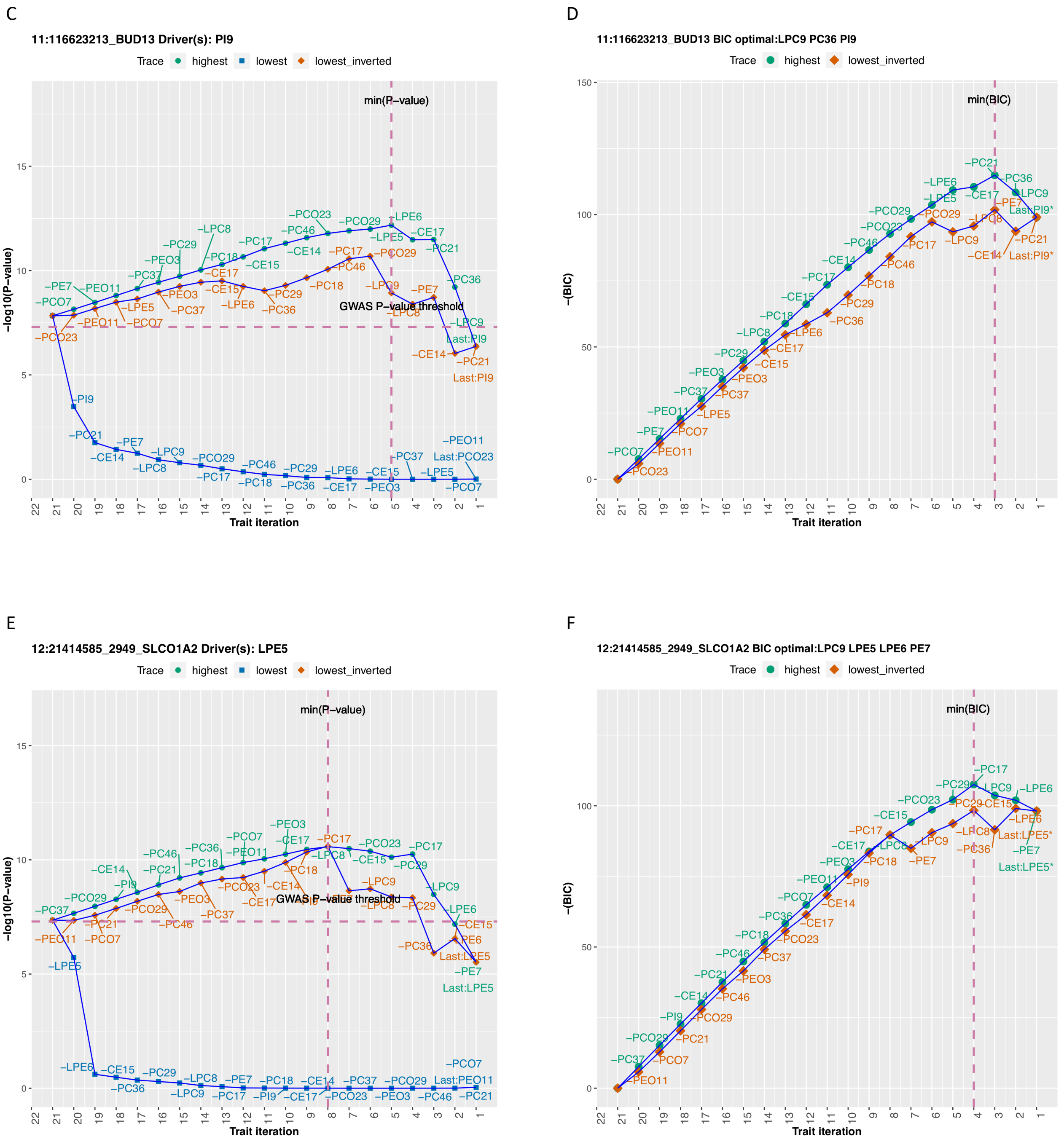
C
11:116623213_BUD13 Driver(s): PI9
Trace highest lowest lowest_inverted
min(P−value)
GWAS P−value threshold
−log10(P−value)
Trait iteration
D
11:116623213_BUD13 BIC optimal:LPC9 PC36 PI9
Trace highest lowest_inverted
min(BIC)
−(BIC)
Trait iteration
E
12:21414585_2949_SLCO1A2 Driver(s): LPE5
Trace highest lowest lowest_inverted
min(P−value)
GWAS P−value threshold
−log10(P−value)
Trait iteration
F
12:21414585_2949_SLCO1A2 BIC optimal:LPC9 LPE5 LPE6 PE7
Trace highest lowest_inverted
min(BIC)
−(BIC)
Trait iteration

G

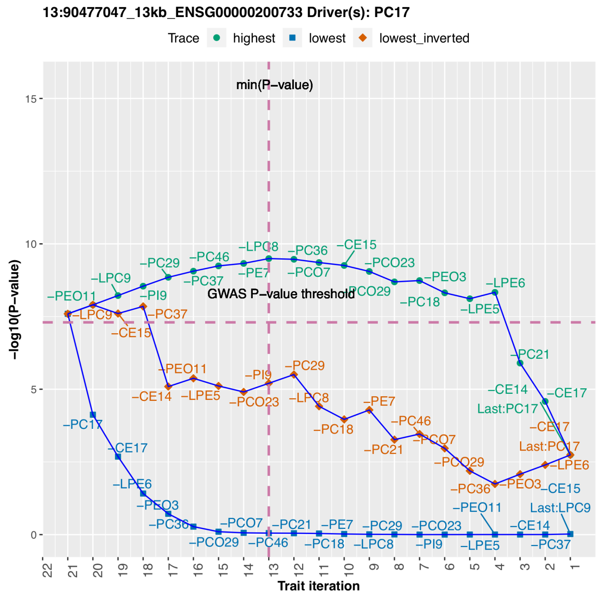
13:90477047_13kb_ENSG00000200733 Driver(s): PC17
Trace highest lowest lowest_inverted
min(P–value)
GWAS P–value threshold
–log10(P–value)
Trait iteration

H

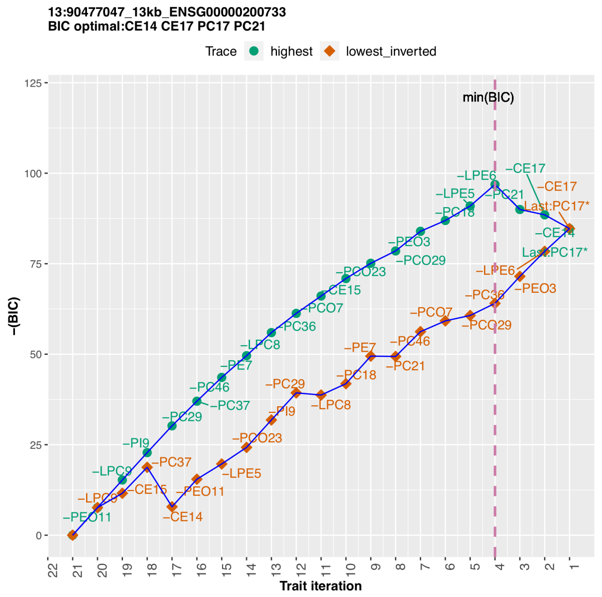
13:90477047_13kb_ENSG00000200733
BIC optimal:CE14 CE17 PC17 PC21
Trace highest lowest_inverted
min(BIC)
–(BIC)
Trait iteration

I

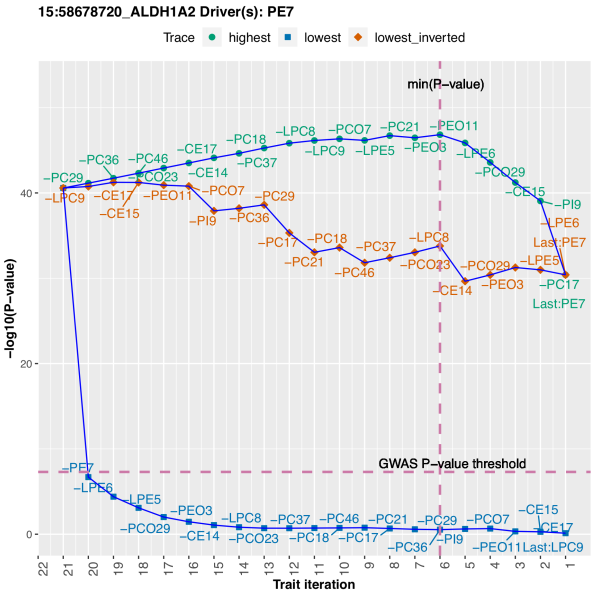
15:58678720_ALDH1A2 Driver(s): PE7
Trace highest lowest lowest_inverted
min(P–value)
GWAS P–value threshold
–log10(P–value)
Trait iteration

J

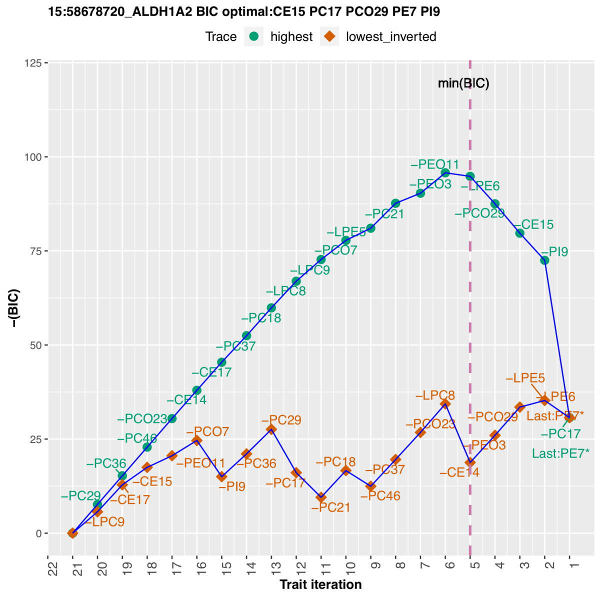
15:58678720_ALDH1A2 BIC optimal:CE15 PC17 PCO29 PE7 PI9
Trace highest lowest_inverted
min(BIC)
–(BIC)
Trait iteration

K

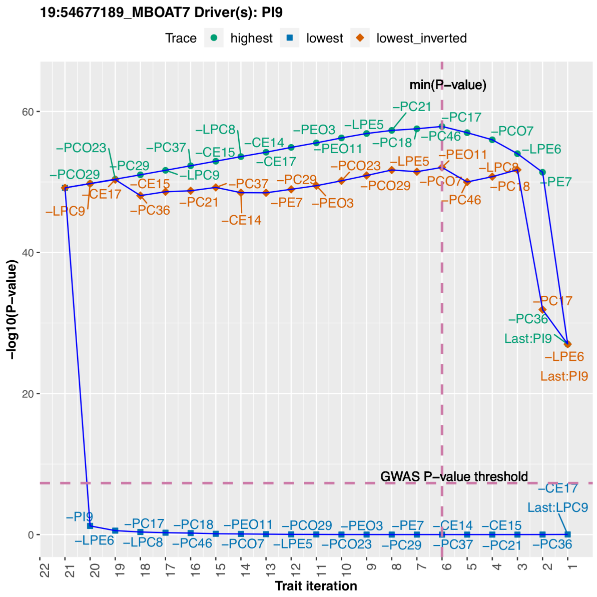

L

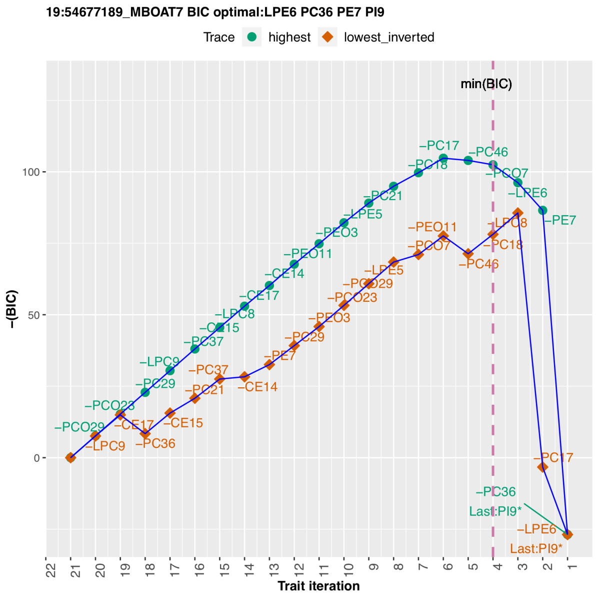

Figure S1 – P-value and BIC trace plots of lead SNP associations detected by MetaPhat decomposition. Additional details are listed in Table 3 of the main text.

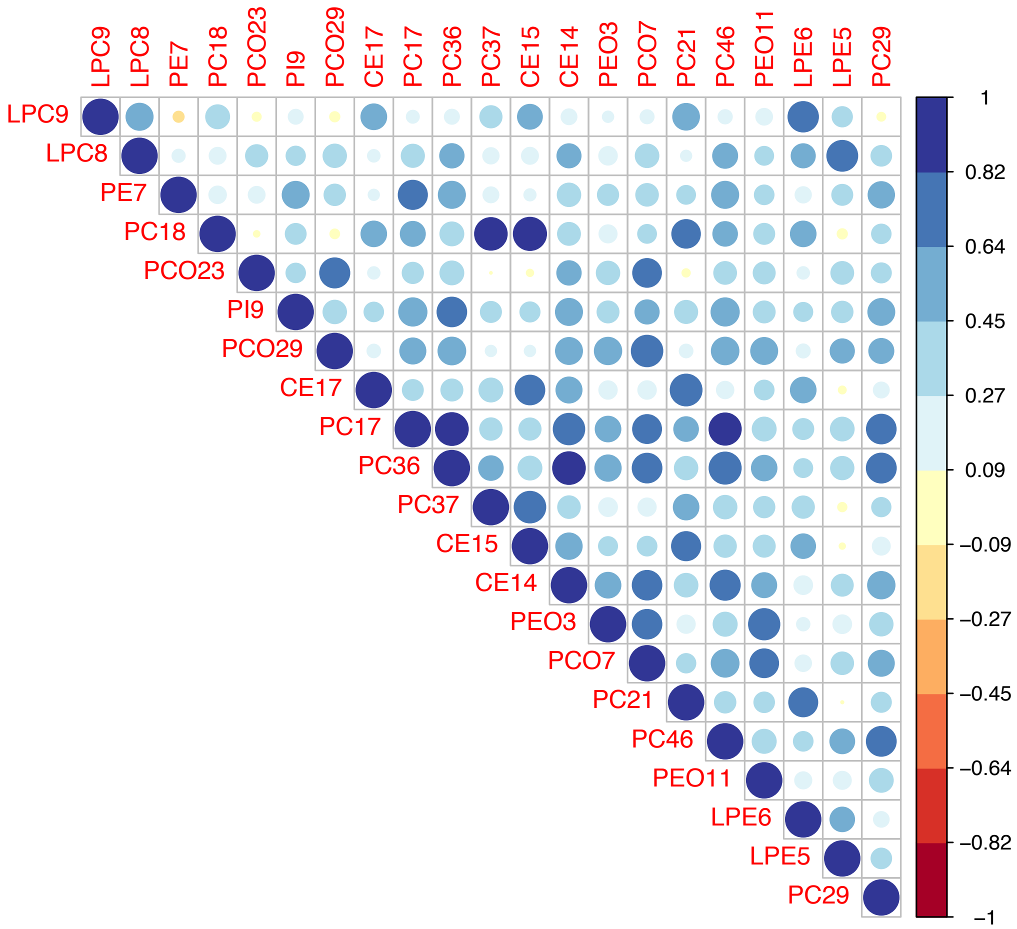

Figure S2: Correlation map of 21 heritable lipid traits computed from univariate beta coefficients using metaCCA.

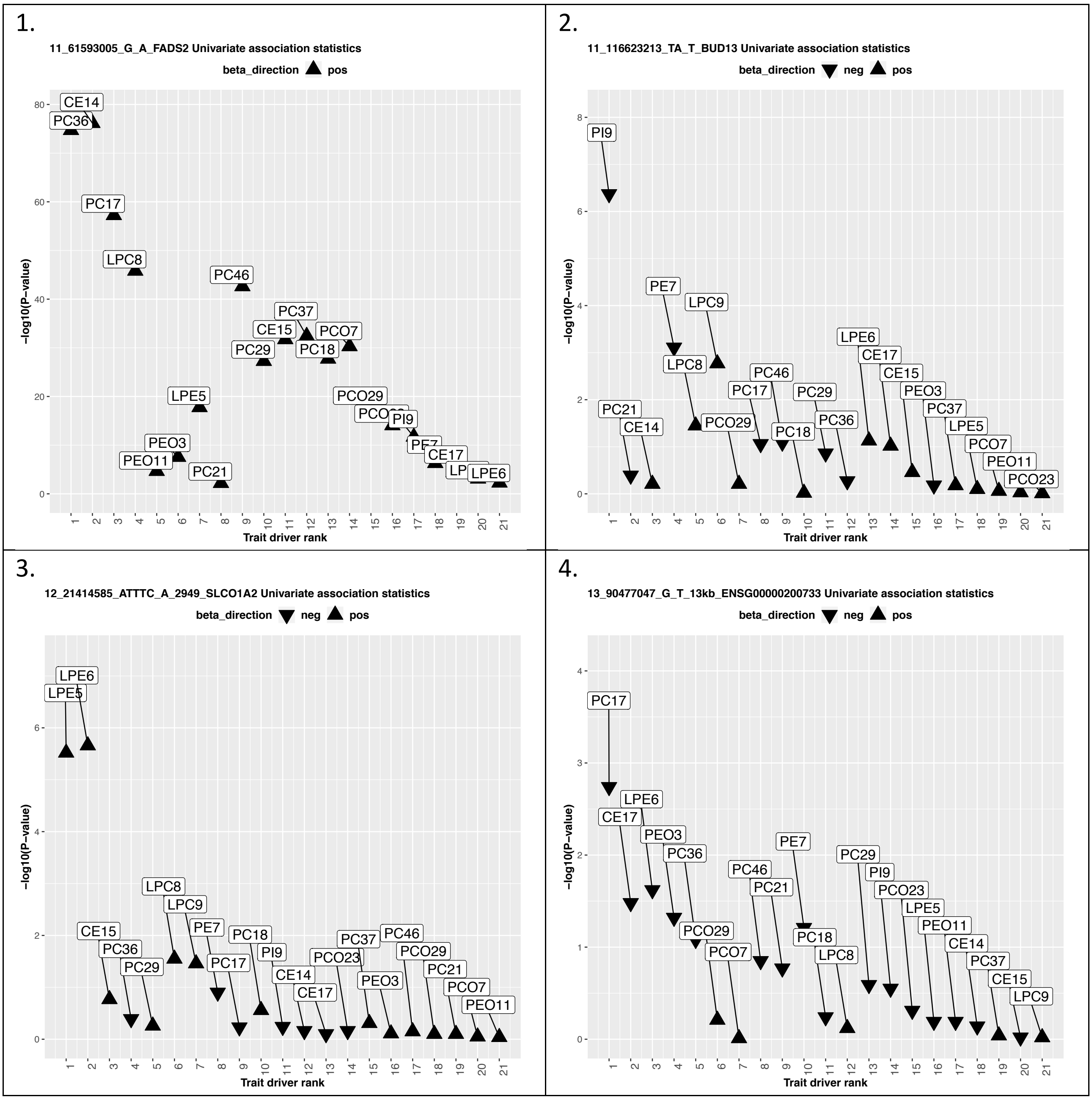
1.
11_61593005_G_A_FADS2 Univariate association statistics
beta_direction pos
-log10(P-value)
Trait driver rank
2.
11_116623213_TA_T_BUD13 Univariate association statistics
beta_direction neg pos
-log10(P-value)
Trait driver rank
3.
12_21414585_ATTTC_A_2949_SLCO1A2 Univariate association statistics
beta_direction neg pos
-log10(P-value)
Trait driver rank
4.
13_90477047_G_T_13kb_ENSG00000200733 Univariate association statistics
beta_direction neg pos
-log10(P-value)
Trait driver rank

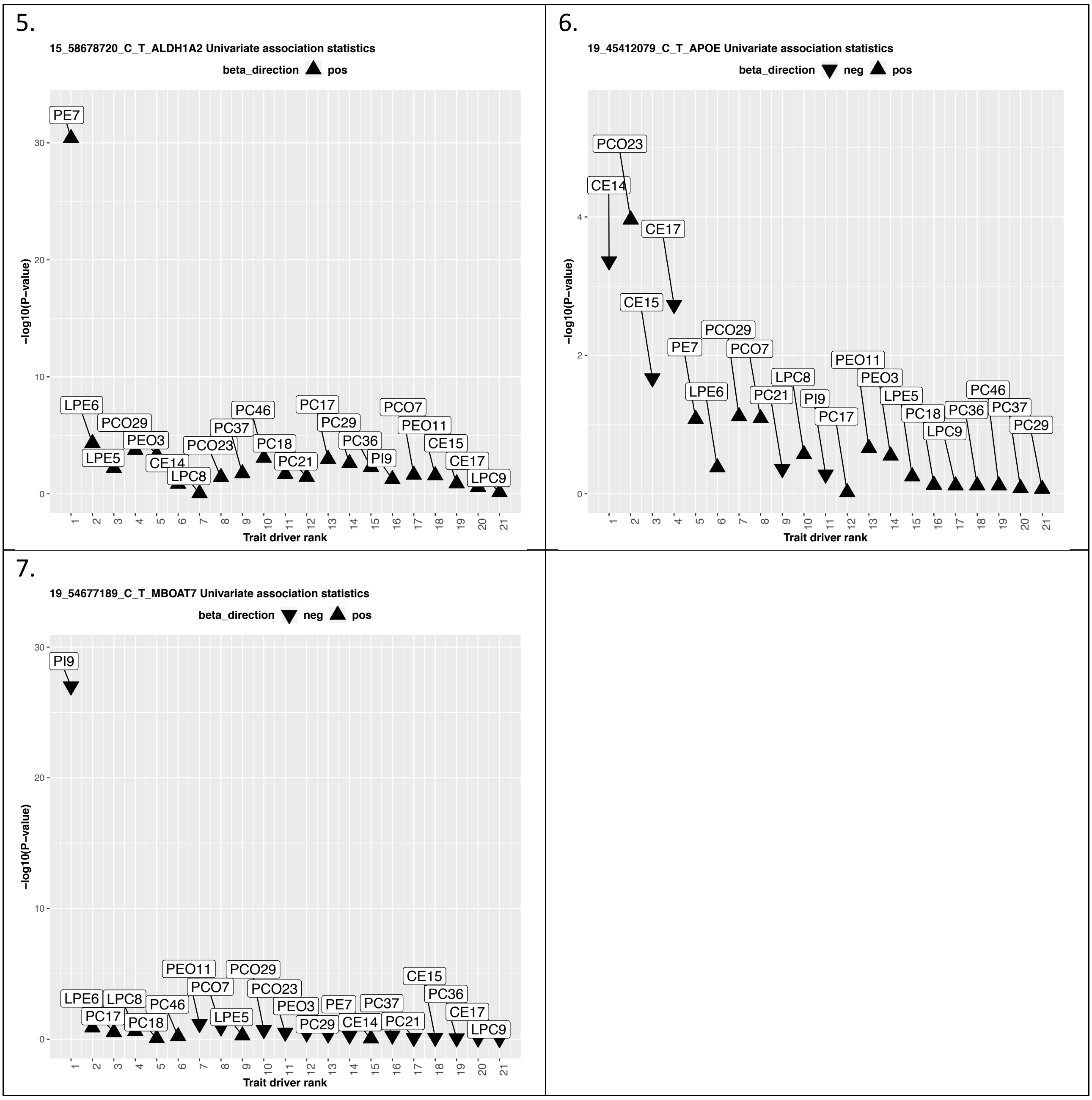

Figure S3: Univariate fatty acid P-value and beta signs of the 7 lead variants are plotted and ordered by the rankings derived from the rank of MetaPhat driver traits.
1. rs174567/FADS2 2. rs66505542/BUD13 3. rs146327691/SLCO1A2_UTR
4. rs188167837/ENSG00000200733_UTR_13KB 5. rs261290/ALDH1A2
6. rs7412/APOE 7. rs8736/MBOAT7

Table S1 – MetaPhat detection of driver and optimal lipids for 69 variants reported to associate with two or more essential GLGC lipids (Willer et al., 2013). As two variants[1] are not included in the summary statistics, the list of 67 variants are group by the reported leading trait, and listed with their central traits. We confirmed that 60/67 of the associated GLGC lipids were either identical or a subset of the central traits.

Gene	VariantId Chr:Pos	GLGC associated Lipids	GLGC Lead P-value	MetaPhat All traits P-value	MetaPhat Driver(s)	Driver(s) Dropped P-value	BIC Optimal set	Central traits
		**HDL lead**						
PIGV-NR0B2	rs731839 chr1:27138393	HDL LDL TG	1e-15	2.8e-23	HDL LDL TG	3.0e-06	HDL LDL	HDL LDL TG
RSPO3	rs1936800 chr6:127436064	HDL TG	3e-10	6.6e-09	TG	7.9e-08	HDL	HDL TG
MARCH8-ALOX5	rs970548 10:46013277	HDL TC	2e-10	1.8e-17	HDL TC	9.1e-04	HDL TC	HDL TC
FTO	rs1121980 chr16:53809247	HDL TG	7e-09	4.9e-09	TG	1.6e-07	HDL	HDL TG
GALNT2	rs4846914 chr1:230295691	HDL TG	4e-41	5.3e-51	HDL TG	1.1e-04	HDL TG	HDL TG
IRS1	rs2972146 chr2:227100698	HDL TG	2e-17	4.6e-22	HDL TG	4.6e-01	HDL TG	HDL TG
PPP1R3B	rs9987289 chr8:9183358	HDL LDL TC	2e-41	1.6e-76	HDL TC LDL	1.0e-04	HDL LDL	ALL
TTC39B	rs581080 chr9:15305378	HDL TC	1e-19	6.9e-46	HDL TC	5.6e-08	HDL LDL TG	ALL
ABCA1	rs1883025 chr9:107664301	HDL TC	2e-65	1.9e-131	HDL TC TG	2.9e-07	HDL LDL TG	ALL
ZNF664	rs4765127 chr12:124460167	HDL TG	8e-10	1.2e-12	HDL TG	1.6e-03	HDL TC	HDL TC TG
LIPC (ALDH1A2)	rs1532085 chr15:58683366	HDL TC TG	1e-188	0	HDL TC LDL	6.4e-01	HDL TG LDL	ALL
CETP	rs3764261 chr16:56993324	ALL	1e-769	0	ALL	1.6e-25	HDL LDL TG	ALL
LIPG	rs7241918 chr18:47160953	HDL TC	1e-44	5.4e-59	HDL TC	9.5e-04	HDL LDL TG	ALL
HNF4A	rs1800961 chr20:43042364	HDL TC	2e-34	1.5e-60	HDL TC LDL	7.1e-01	HDL LDL TG	ALL
PLTP (ZNF335)	rs6065906 chr20:44554015	HDL TG	5e-40	8.9e-50	HDL TG	1.3e-03	HDL TG	HDL TG
		**LDL lead**						
INSIG2	rs10490626 2:118835841	LDL TC	2e-12	9.8e-14	TG LDL HDL	5.3e-02	TG LDL	LDL TG
LOC84931	rs2030746 2:121309488	LDL TC	9e-09	5.9e-07	NA	NA	LDL	LDL
CMTM6	rs7640978 3:32533010	LDL TC	1e-08	1.8e-08	LDL	1.4e-07	LDL	LDL
ACAD11 (DNAJC13)	rs17404153 chr3:132163200	LDL HDL	2e-09	1.8e-08	LDL	1.2e-07	LDL	LDL
CSNK1G3	rs4530754 5:122855416	LDL TC	4e-12	1.7e-12	LDL TC	7.9e-01	LDL	LDL TC
MIR148A	rs4722551 7:25991826	LDL TG TC	4e-14	2.5e-24	TG LDL TC	2.0e-02	LDL TG	LDL TG TC
SOX17	rs10102164 8:55421614	LDL TC	4e-11	5.0e-12	LDL TC	1.0e-02	LDL	LDL TC
PCSK9	rs2479409 1:55504650	LDL TC	3e-50	3.2e-53	LDL TC	2.8 e-02	LDL	LDL TC

SORT1	rs629301 1:109818306	LDL TC	5e-241	1.3e-270	LDL TC TG	6.3e-01	LDL TG	LDL TC TG
APOB	rs1367117 2:21263900	ALL	1e-182	5.0e-197	ALL	7.9e-09	LDL TC	ALL
ABCG5	rs4299376 2:44072576	LDL TC	4e-72	1.0e-79	LDL TC	6.3e-03	LDL TC	LDL TC
MYLIP	rs3757354 6:16127407	LDL TC	1e-17	1.3e-17	LDL TC	6.3e-01	LDL	LDL TC
HFE	rs1800562 6:26093141	LDL TC	8e-14	1.6e-15	TG LDL TC	2.4e-01	LDL	LDL TG TC
LPA	rs1564348 6:160578860	LDL TC	3e-21	1.3e-23	LDL TC	1.2e-03	TC	LDL TC
PLEC1	rs11136341 8:145043543	LDL TC	7e-12	1.0e-11	LDL TC	6.3e-01	LDL	LDL TC
ST3GAL4	rs11220462 11:126243952	LDL TC	7e-21	6.3e-22	LDL TC	7.9e-04	LDL	LDL TC
OSBPL7	rs7206971 17:45425115	LDL TC	1e-07	1e-09	HDL TG LDL	3.2e-07	LDL HDL	ALL
LDLR	rs6511720 19:11202306	LDL TC	4e-262	5.0e-296	LDL TC	6.3e-05	LDL TG	LDL TC TG
APOE (APOC)	rs4420638 19:45422946	LDL TC HDL	2e-178	6.3e-210	ALL	1.6e-14	LDL HDL TC	ALL
TOP1	rs6029526 20:39672618	LDL TC	5e-18	1.0e-15	LDL TC	1.1e-01	LDL	LDL TC
		**TC Lead**						
UGT1A1	rs11563251 2:234679384	TC LDL	6e-09	2.0e-08	TC LDL	5.0e-02	TC	TC LDL
VLDLR	rs3780181 9:2640759	TC LDL	7e-10	6.3e-10	TC LDL	5.1e-01	TC	TC LDL
DLG4	rs314253 17:7091650	TC LDL	3e-10	1.6e-09	LDL TC	3.9e-02	LDL	TC LDL
PPARA	rs4253772 22:46627603	TC LDL	1e-08	1.9e-08	TG	3.2e-07	LDL	LDL TG
LDLRAP1	rs12027135 1:25775733	TC LDL	5e-12	4.0e-14	TC LDL	6.3e-02	LDL	TC LDL
MOSC1 (MARC1)	rs2642442 1:220973563	TC LDL	3e-11	2.5e-17	LDL TC	1.3e-05	HDL LDL	TC LDL HDL
IRF2BP2	rs514230 1:234858597	TC LDL	5e-14	1.3e-11	TC LDL	1.6e-01	TC	TC LDL
HMGCR	rs12916 5:74656539	TC LDL	5e-74	1.6e-90	TC LDL	2.0e-01	TC LDL	TC LDL
TIMD4	rs6882076 5:156390297	TC LDL TG	5e-41	1.3e-49	TG TC LDL	6.9e-01	HDL LDL TG	ALL
HLA	rs3177928 6:32412435	TC LDL	1e-21	2.0e-24	HDL TC LDL	3.2e-03	HDL LDL TG	ALL
FRK	rs9488822 6:116312893	TC LDL	1e-09	1.3e-10	TG TC LDL	2.0e-01	LDL	TC TG LDL
DNAH11	rs12670798 7:21607352	TC LDL	1e-16	6.3e-17	TC LDL	2.5e-02	TC	TC LDL
NPC1L1	rs2072183 7:44579180	TC LDL	4e-15	7.9e-16	TC LDL	4.0e-03	LDL	TC LDL
CYP7A1	rs2081687 8:59388565	TC LDL	9e-12	5.0e-13	TG TC LDL	5.0e-01	TC	TC TG LDL
GPAM	rs2255141 10:113933886	TC LDL	7e-16	1.0e-36	ALL	1.3e-13	HDL LDL	ALL
BRAP	rs11065987 12:112072424	TC LDL	2e-16	7.9e-24	TC LDL TG	2.0e-01	LDL HDL	ALL

HNF1A	rs1169288 12:121416650	TC LDL	4e-17	2.5e-23	LDL TC	4.0e-03	LDL HDL	TC LDL HDL
HPR	rs2000999 16:72108093	TC LDL	7e-41	1.6e-49	LDL TC	1.3e-06	LDL TC	TC LDL
CILP2	rs10401969 19:19407718	TC LDL TG	4e-77	1.3e-138	TG HDL TC	1.0e-01	HDL LDL TG	ALL
MAFB	rs2902940 20:39091487	TC LDL	9e-10	2.5e-11	TC LDL	1.8e-02	LDL	TC LDL
		**TG Lead**						
LRPAP1 (DOK7)	rs6831256 4:3473139	TG TC LDL	2e-12	6.3e-16	TG TC	1.0e-07	TG TC	TG TC
VEGFA	rs998584 6:43757896	TG HDL	3e-15	1.2e-16	TG HDL	5.3e-01	HDL	TG HDL
PEPD	rs731839 19:33899065	TG HDL	3e-09	2.5 e-11	TG HDL	1.3e-01	TG	TG HDL
ANGPTL3	rs2131925 1:63025942	TG LDL TC	3e-74	7.8e-157	TG TC LDL	9.5e-05	TG TC HDL	TG LDL TC
GCKR	rs1260326 2:27730940	TG TC	2e-239	2.0e-290	TG TC LDL	1.7e-03	TG TC HDL	TG TC LDL
MLXIPL	rs17145738 7:72982874	TG HDL	9e-99	7.9e-105	TG HDL	7.8e-06	TG	TG HDL
NAT2	rs1495741 8:18272881	TG TC	3e-12	5.9e-14	TG	1.1e-07	TG	TG
LPL	rs12678919 8:19844222	TG HDL	2e-199	1.0e-253	TG HDL	9.5e-02	TG LDL HDL	TG HDL LDL
TRIB1	rs2954029 8:126490972	ALL	1e-107	1.6e-148	ALL	NA	TG LDL	ALL
FADS123	rs174546 11:61569830	ALL	7e-38	1.3e-104	ALL	NA	ALL	ALL
APOA1	rs964184 11:116648917	ALL	7e-224	7.9e-264	ALL	NA	TG TC	ALL
LRP1	rs11613352 12:57792580	TG HDL	9e-14	7.9e-16	TG HDL	1.6e-04	TG HDL	TG HDL

Two associations not found in lipid summaries: ABO (rs7941030) LDL TC and UBASH3B (rs7941030):TC HDL

Table S2: The input switches of MetaPhat are shown with the --help option. Table below outlines the argument switches and their default values.

Switches	Description and default value
--help	Shows all arguments and lists relevant default values
--phenotypes	Required, parameter is a file that defines GWAS summary files for testing (key:full_path)<br><br>One summary file per line, in this format<br>pheno1:/path/summary1.gz<br>pheno2:/path/summmary2.gz<br>…<br>See example:<br>https://sourceforge.net/projects/meta-pheno-association-tracer/files/test_inputs/global_lipid_summaries2
--outlabel	Required, this defines a label for output files and plotting, must be alphanumeric and without spaces.
--nsamples	Required, enter in estimate number of subjects the GWAS summaries were based on.
--nsamples_dev	Optional, this defines the allowed deviating percentage of missing samples for variants to be included in analysis. It only applies if N is defined in

	GWAS summaries. Defaults to .25 (25%, for example, if sample size N =1000, variants with <750 or >1250 will be excluded)
--chunksize	Required, this sets the number of SNPs processed by metaCCA on each batch. Defaults to 100000
--parallel	Required, this defines the number of threads, or batch jobs executed at the same time. The entry depends on your processor and whether it is shared. Defaults to 5.
--waittime	Required, this defines the number of seconds each batch submission approximately runs. Waittime is important and depends on the chunksize and parallel threads, our testing is that this should be around 120-180 seconds for chunks of 100000 and 6-8 threads on 16Gb 16Core nonshared environment. On shared server, we recommend using higher wait time. Defaults to 180.
--r1	Optional, this defines the output column from metaCCA to sort by during clumping. metaCCA outputs r1 and pvalue and we recommend r1 as pvalue can be INF and problematic for true sort of importance. Defaults to 1 meaning CCA correlation.
--outdir	Required, this defines the complete path to store outputs.
--gwascutoff	Required. This defines the pvalue cutoff, -1(math.log(pv, 10)) Defaults to 7.3, (5.0e-8) approximating recommended GWAS threshold
--grch	Optional, this defines the human ENSEMBL (Hunt et al. 2018) build version, possible values are 37 and 38. Defaults to 37.
--exclude	Optional, this defines a file where variants can be excluded even if the variant is significant. One variant per line and see example: https://sourceforge.net/projects/meta-pheno-association-tracer/files/test_inputs/exclude.dat
--interested	Optional, this defines a file where variants are included in the trace results even if variant pvalue does not pass cutoff. One variant per line and see example: https://sourceforge.net/projects/meta-pheno-association-tracer/files/test_inputs/interested.list
--maf_min	Optional, this defines the minor allele frequency minimum cutoff. Defaults to 0.01. MAF is derived from the related effective allele frequency (EAF). MAF is set to EAF if EAF is < 0.50, otherwise, MAF = 1 – EAF.<br>For possible guidelines, see UK Biobank (item 2): http://www.nealelab.is/blog/2017/9/11/details-and-considerations-of-the-uk-biobank-gwas
--clump	Required, this defines the base window to sort variants and their pvalues/r1 canonical correlation. Defaults to 500000.
--neglogval	Optional. Parameter defines the maximal neg log pvalue for INF values. Defaults to 400.
--plot_intermediate	Optional. Parameter tells program to plot intermediate lead cca value for all iterations. Defaults to 0.
--title_font	Optional. Parameter defines the font size of title in trace plot. Defaults to 12.
--element_font	Optional. Parameter defines the font size of element label in trace plot. Defaults to 5.
--axis_font	Optional. Parameter defines the font size of axis label in trace plot. Defaults to 12.
--legend_font	Optional. Parameter defines the font size of legend in trace plot. Defaults to 12.
--Rscript	Required, this defines the full path to Rscript executable. Defaults to /usr/bin/Rscript
